# Two new forest pathogens in *Phaeolus* (Polyporales, Basidiomycota) on Chinese coniferous trees were confirmed by molecular phylogeny

**DOI:** 10.3389/fmicb.2022.942603

**Published:** 2022-09-21

**Authors:** Yuan Yuan, Ying-Da Wu, Ya-Rong Wang, Meng Zhou, Jun-Zhi Qiu, De-Wei Li, Josef Vlasák, Hong-Gao Liu, Yu-Cheng Dai

**Affiliations:** ^1^Institute of Microbiology, School of Ecology and Nature Conservation, Beijing Forestry University, Beijing, China; ^2^Key Laboratory of Forest and Grassland Fire Risk Prevention, Ministry of Emergency Management, China Fire and Rescue Institute, Beijing, China; ^3^College of Life Science, Fujian Agriculture and Forestry University, Fuzhou, China; ^4^The Connecticut Agricultural Experiment Station Valley Laboratory, Windsor, CT, United States; ^5^Biology Centre, Institute of Plant Molecular Biology, Czech Academy of Sciences, České Budějovice, Czechia; ^6^School of Agronomy and Life Sciences, Zhaotong University, Zhaotong, China

**Keywords:** brown rot, pathogenetic fungi, phylogeny, polypore, wood-decaying fungi

## Abstract

*Phaeolus schweinitzii* (Fr.) Pat. was originally described in Europe and is considered a common forest pathogen on conifers in the Northern Hemisphere. Our molecular phylogeny based on samples from China, Europe, and North America confirms that *P. schweinitzii* is a species complex, including six taxa. *P. schweinitzii* sensu stricto has a distribution in Eurasia; the samples from Northeast and Southwest China are distantly related to *P. schweinitzii* sensu stricto, and two new species are described after morphological, phylogenetic, and geographical analyses. The species growing on *Larix, Picea*, and *Pinus* in Northeast China is described as *Phaeolus asiae-orientalis*. Another species mostly occurring on *Pinus yunnanensis* in Southwest China is *Phaeolus yunnanensis*. In addition, three taxa distributed in North America differ from *P. schweinitzii* sensu stricto. *Phaeolus tabulaeformis* (Berk.) Pat. is in Southeast North America, “*P. schweinitzii*-1” in Northeast North America, and “*P. schweinitzii*-2” in western North America.

## Introduction

*Phaeolus* (Pat.) Pat. is a well-known polypore genus because of its type species; *Phaeolus schweinitzii* (Fr.) Pat. has conspicuous and colorful basidiocarps and is the major cause of butt rotting in many commercial timber species (Gilbertson and Ryvarden, 1987; Sinclair et al., 1987; Núñez and Ryvarden, 2001; Ryvarden and Melo, 2017). The genus is characterized by annual, pileate to stipitate, orange to brown basidiocarps with a fibrous to spongy context, a monomitic hyphal system with simple septate hyphae, the presence of gloeoplerous hyphae and cystidia, ellipsoid to cylindric, hyaline, thin-walled, acyanophilous, neither amyloid nor dextrinoid basidiospores, and causing brown rot. According to current taxonomy, *Phaeolus* is closely related to *Wolfiporia* Ryvarden & Gilb. and belongs to the family Laetiporaceae in the order Polyporales (Justo et al., 2017; Wu et al., 2020).

Although many epithets are listed in *Phaeolus*, most were treated as synonyms of *P. schweinitzii* (Donk, 1974; Index Fungorum: http://www.indexfungorum.org/names/Names.asp, MycoBank: https://www.mycobank.org/page/Simple%20names%20search). Previously, five species only, viz., *Phaeolus amazonicus* M.A. De Jesus & Ryvarden, *P. subbulbipes* (Henn.) O. Fidalgo & M. Fidalgo, *P. manihotis* R. Heim, *P. tabulaeformis* (Berk.) Pat., and *P. schweinitzii* (Fr.) Pat., were accepted in the genus. The former three species were described from Brazil and Madagascar, respectively, with a limited distribution (Heim, 1931; Fidalgo and Fidalgo, 1957; Jesus and Ryvarden, 2010), and *P. tabulaeformis* was described from Georgia, USA (Patouillard, 1900). *P. schweinitzii* was described from Europe and reported to be widely distributed in The Northern Hemisphere (Donk, 1974; Gilbertson and Ryvarden, 1987; Núñez and Ryvarden, 2001; Ryvarden and Melo, 2017). *P. schweinitzii* was also reported in Australia and New Zealand (Buchanan and Ryvarden, 2000; Simpson and May, 2002).

During investigations on brown-rot fungi in China, specimens morphologically similar to *P. schweinitzii* were collected from Northeast (NE) and Southwest (SW) China, but they are different from the European *P. schweinitzii* based on our preliminary phylogenetic study. Thus, it appears that *P. schweinitzii* is a species complex with more independent species that exist in the Northern Hemisphere. Then, more samples from North America and Europe were added for the multigene phylogeny, and six independent lineages were formed. After morphological examinations and phylogenetic and geographical analyses, six taxa are recognized in the *P. schweinitzii* complex; two taxa are confirmed as new members of *Phaeolus*, and they are pathogens on coniferous trees in NE and WS China. The present study aims to clarify the Chinese species of *Phaeolus* and outline the phylogeny of *Phaeolus* based on available data in the Northern Hemisphere.

## Materials and methods

### Morphological studies

The examined specimens are deposited at the herbaria of the Institute of Microbiology, Beijing Forestry University (BJFC), and the Institute of Applied Ecology, Chinese Academy of Sciences (IFP). Macro-morphological descriptions were based on field notes and measurements of voucher herbarium specimens. Microscopic measurements and drawings were obtained from the slides prepared from voucher specimens and stained with Cotton Blue and Melzer's reagent following Wu et al. (2022) using a Nikon Eclipse 80i microscope. The following abbreviations were used in the description: CB = Cotton Blue, CB– = acyanophilous, IKI = Melzer's reagent, IKI– = neither amyloid nor dextrinoid, L = mean spore length (arithmetic average of spores), W = mean spore width (arithmetic average of spores), *Q* = variation in the L/W ratios between specimens studied, *n* (a/b) = number of spores (a) measured from a given number of specimens (b). In presenting spore size variation, mean ± SD (standard deviation at 95% confidence) was reported as the range; 5% of measurements were excluded from each end of the range, and the values are given in parentheses. Special color terms follow Anonymous (1969) and Petersen (1996). Herbarium abbreviations follow Thiers (2022).

### DNA extraction and sequencing

The Rapid Plant Genome kit based on acetyl trimethylammonium bromide extraction (Aidlab Biotechnologies Co., Ltd, Beijing, China) was used to extract total genomic DNA from dried specimens and for polymerase chain reaction (PCR), according to the manufacturer's instructions with some modifications (Song and Cui, 2017; Xing et al., 2018). The ITS region was amplified with the primer pairs ITS5 and ITS4 (White et al., 1990). The nLSU region was amplified with primer pairs LR0R and LR7 (http://www.biology.duke.edu/fungi/mycolab/primers.htm). The PCR procedure was followed by Yuan et al. (2021). The PCR products were purified and sequenced at the Beijing Genomics Institute, China, with the same primers as in the original PCR amplifications.

### Phylogenetic analyses

**Figure 1:**
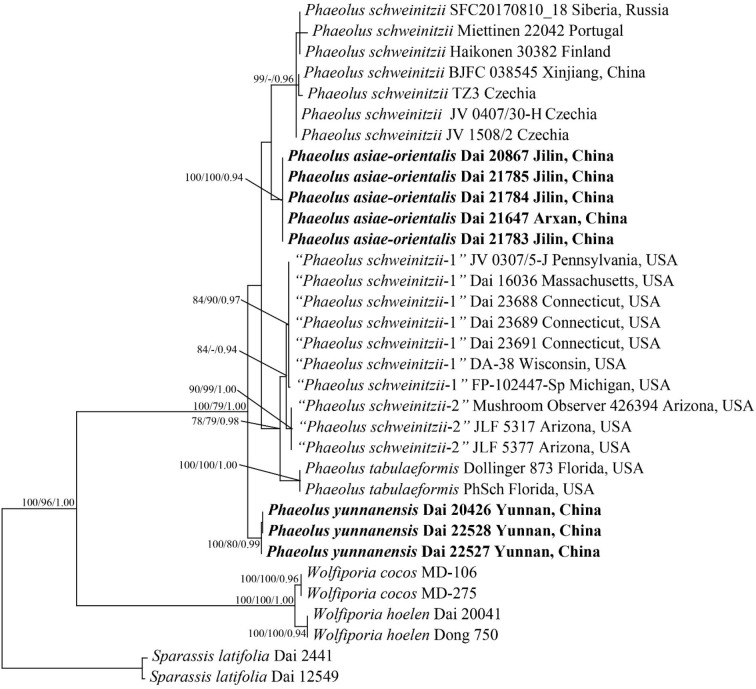
Phylogeny of *Phaeolus* generated by maximum parsimony based on a dataset of ITS + nLSU. Branches are labeled with bootstrap values (MP/ML) higher than 75% and posterior probabilities (BI) more than 0.90, respectively. New taxa are in bold.

The phylogenetic tree was constructed using sequences obtained in this study and additional sequences downloaded from GenBank (Table 1). The sequences were aligned within MAFFT version 7 (Katoh et al., 2019) and ClustalX (Thompson et al., 1997), then manual proofreading was performed in BioEdit (Hall, 1999). The downloaded sequences were chosen to cover Laetiporaceae Jülich and related clades, including Fomitopsidaceae Jülich and Sparassidaceae Herter (Justo et al., 2017; Song and Cui, 2017; Song et al., 2018). Ambiguous regions were deleted, and gaps were manually adjusted to optimize alignment before phylogenetic analyses. *Sparassis latifolia* Y. C. Dai and Zheng Wang was used as an outgroup in the phylogeny of *Phaeolus* (Zhao et al., 2013; Figure 1). The data matrix was edited in Mesquite version 3.04 software. Phylogenetic analyses were performed with maximum parsimony (MP), maximum likelihood (ML), and Bayesian Inference (BI) based on ITS + nLSU aligned datasets.

**Table 1:** Taxa information and GenBank accession numbers of the sequences used in this study.

**Species**	**Collection**	**Geographic origin**	**Host**	**GenBank accessions**	
				**ITS**	**nLSU**
***Phaeolus asiae-orientalis***	**Dai 20867**	**Jilin, China**	***Picea***	**ON310980**	**ON310967**
***P. asiae-orientalis***	**Dai 21647**	**Arxan, China**	***Larix***	**ON310981**	**–**
***P. asiae-orientalis***	**Dai 21783**	**Jilin, China**	***Pinus***	**ON310982**	**ON310968**
***P. asiae-orientalis***	**Dai 21784**	**Jilin, China**	***Pinus***	**ON310983**	**ON310969**
***P. asiae-orientalis***	**Dai 21785**	**Jilin, China**	***Pinus***	**ON310984**	**ON310970**
*P. schweinitzii*	BJFC 038545	Xinjiang, China	*Larix*	MW551143	MW520024
***P. schweinitzii***	**Haikonen 30382**	**Finland**	***Larix***	**ON310986**	**ON310972**
***P. schweinitzii***	**JV0407/30-H**	**Czechia**	***Pinus***	**ON310987**	**–**
***P. schweinitzii***	**JV1508/2**	**Czechia**	***Prunus***	**ON310988**	**–**
***P. schweinitzii***	**Miettinen 22042**	**Portugal**	***Pinus pinea***	**ON310985**	**ON310971**
*P. schweinitzii*	SFC20170810_18	Siberia, Russia	–	MT044415	–
*P. schweinitzii*	TZ3	Czechia	–	LN714583	–
***P. tabulaeformis***	**Dollinger 873**	**Florida, USA**		**ON310989**	**–**
*P. tabulaeformis*	PhSch	Florida, USA	–	MW795374	–
***P. yunnanensis***	**Dai 20426**	**Yunnan, China**	***Pinus yunnanensis***	**ON310995**	**ON310977**
***P. yunnanensis***	**Dai 22527**	**Yunnan, China**	***Pinus yunnanensis***	**ON310996**	**ON310978**
***P. yunnanensis***	**Dai 22528**	**Yunnan, China**	***Pinus yunnanensis***	**ON310997**	**ON310979**
“*P. schweinitzii*-1”	DA-38	Wisconsin, USA	*Prunus serotina*	EU402585	EU402514
***“P. schweinitzii*** **-1”**	**Dai 16036**	**Massachusetts, USA**	***Tsuga***	**ON310991**	**ON310976**
***“P. schweinitzii*** **-1”**	**Dai 23688**	**Connecticut, USA**	***Pinus strobus***	**ON310992**	**ON310973**
***“P. schweinitzii*** **-1”**	**Dai 23689**	**Connecticut, USA**	***Pinus strobus***	**ON310993**	**ON310974**
***“P. schweinitzii*** **-1”**	**Dai 23691**	**Connecticut, USA**	***Pinus strobus***	**ON310994**	**ON310975**
“*P. schweinitzii*-1”	FP-102447-Sp	Michigan, USA	*Pinus*	KC585368	KC585197
***“P. schweinitzii*** **-1”**	**JV 0307/5-J**	**Pennsylvania, USA**	***Tsuga***	**ON310990**	**–**
“*P. schweinitzii*-2”	JLF 5317	Arizona, USA	–	MH277963	–
“*P. schweinitzii*-2”	JLF 5377	Arizona, USA	–	MH277964	–
“*P. schweinitzii*-2”	Mushroom Observer 426394	Arizona, USA	*Pinus ponderosa*	OK058472	–
*Sparassis latifolia*	Dai 2441	China	*Larix*	JQ743075	JQ743085
*S. latifolia*	Dai 12549	China	*Larix*	JQ743076	JQ743086
*Wolfiporia cocos*	MD-106	USA	*Alnus*	EU402594	EU402519
*W. cocos*	MD-275	USA	*Pinus*	EU402595	EU402520
*W. hoelen*	Dai 20041	China	*Pinus*	MW251878	MW251867
*W. hoelen*	Dong 750	China	*Pinus*	MW251873	MW251862

New sequences are shown in bold.

MP analysis was applied to the dataset containing the ITS + nLSU sequences. The tree construction procedure was performed using PAUP^*^ version 4.0b10 (Swofford, 2002). All characters were equally weighted, and gaps were treated as missing data. Trees were inferred using the heuristic search option with TBR branch swapping and 1,000 random sequence additions. Max-trees were set to 5,000, branches of zero length were collapsed, and all parsimonious trees were saved. Clade robustness was assessed using a bootstrap analysis with 1,000 replicates (Felsenstein, 1985). Descriptive tree statistics: tree length (TL), consistency index (CI), retention index (RI), rescaled consistency index (RCI), and homoplasy index (HI) were calculated for each maximum parsimonious tree generated.

jModeltest v.2.17 (Darriba et al., 2012) was used to determine the best-fit evolution model of the combined dataset for ML and BI. Four unique partitions were established; GTR + I + G was the selected substitution model for each partition. RAxML version 8.2.12 (Stamatakis, 2014) was used for ML analysis with default parameters. Only the best maximum likelihood tree from all searches was kept.

The BI was calculated with MrBayes version 3.2.6 (Ronquist et al., 2012) in two independent runs, each of which had four chains for 10 million generations and started from random trees. Trees were sampled every 100 generations. The first 25% of sampled trees were discarded as burn-in, whereas other trees were used to construct a 50% majority consensus tree and for calculating Bayesian posterior probabilities (BPPs).

Phylogenetic trees were visualized using TreeView (Page, 1996). Branches that received bootstrap support for Maximum likelihood (BS), Maximum parsimony (BP), and Bayesian posterior probabilities (BPP) ≥75% (BS and BP) and 0.90 (BPP) were considered as significantly supported, respectively.

## Results

### Molecular phylogeny

The ITS + nLSU sequences from 33 fungal collections represent nine species. Among them, 31 new sequences were generated in this study. The dataset had an aligned length of 1,944 characters, of which 1,473 characters are constant, six are variable and parsimony-uninformative, and 465 are parsimony-informative. MP analysis yielded a tree (TL = 626, CI = 0.925, RI = 0.972, RC = 0.899, HI = 0.075). The best model for the ITS + nLSU sequences dataset estimated and applied in the Bayesian analysis was GTR + I + G with an equal frequency of nucleotides, lset nst = 6 rates = invgamma; prset statefreqpr = dirichlet (1,1,1,1). The Bayesian analysis resulted in a similar topology to MP, with an average standard deviation of split frequencies = 0.004684; thus, only the MP tree is displayed (Figure 1). Two new species, *Phaeolus asiae-orientalis* (100% MP, 100% ML, 0.94 BI) and *P. yunnanensis* (100% MP, 80% ML, 1.00 BI), formed well-supported phylogenetic lineages, respectively.

According to the present phylogenetic analyses, the *P. schweinitzii* complex consists of six taxa: *P. schweinitzii* sensu stricto seems to be distributed in Eurasia; *P. asiae-orientalis* and *P. yunnanensis* are so far known in Northeast and Southwest China; *P. tabulaeformis* (Berk.) Pat., “*P. schweinitzii-*1,” and “*P. schweinitzii-*2” have distributions in North America.

### Taxonomy

**Figure 2:**
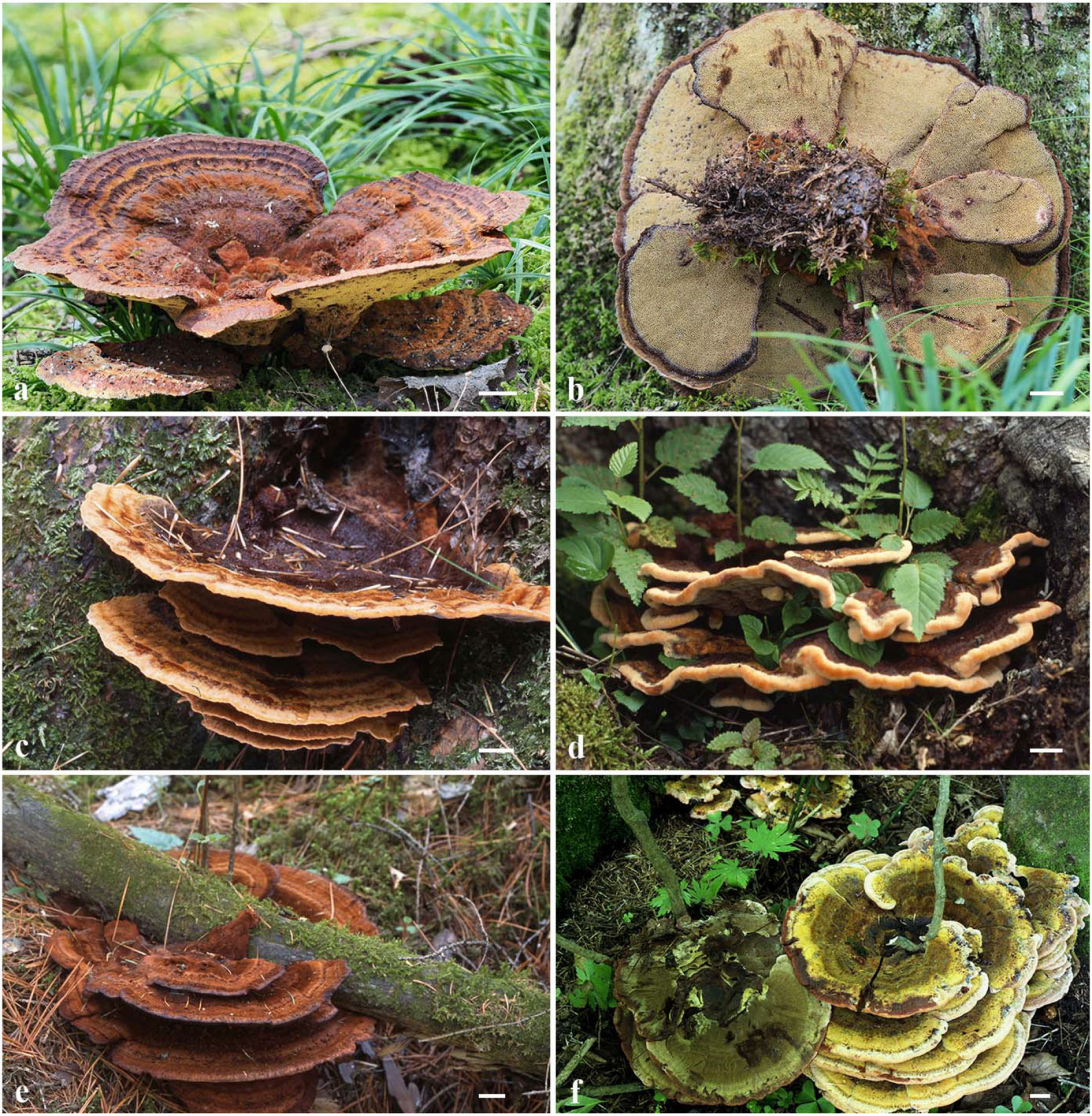
Basidiocarps of *Phaeolus asiae-orientalis*. **(a,b)** Dai 20867 (holotype); **(c)** Dai 789; **(d)** Dai 7089; **(e)** Dai 21784; **(f)** Dai 14566. Scale bars = 1 cm.

**Figure 3:**
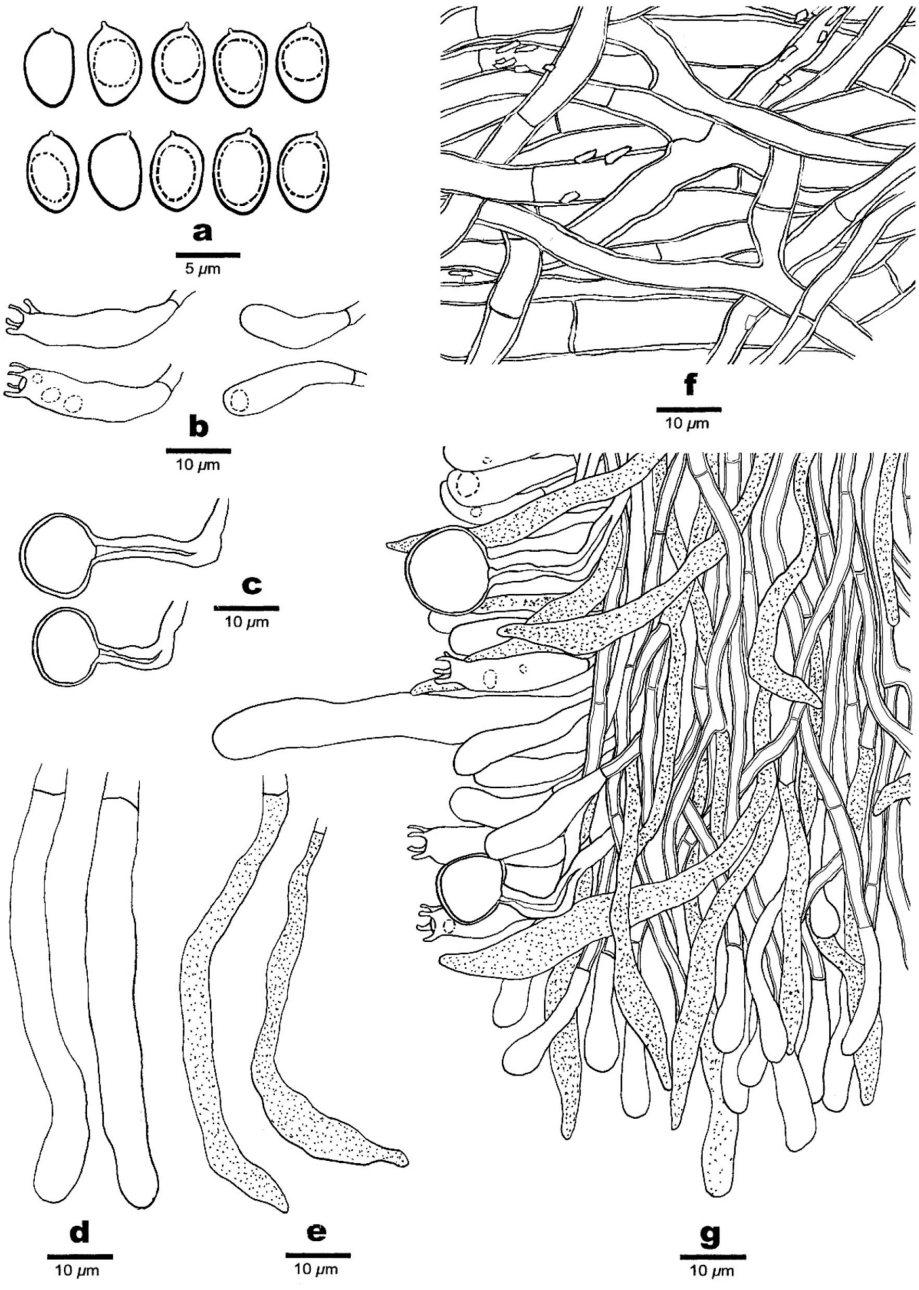
Microscopic structures of *Phaeolus asiae-orientalis* (drawn from the holotype, Dai 20867). **(a)** Basidiospores. **(b)** Basidia and basidioles. **(c)** Vascular elements. **(d)** Cystidia. **(e)** Gloeocystidia. **(f)** Hyphae from context. **(g)** A section from tube trama.

***Phaeolus asiae-orientalis*** Y.C. Dai & Yuan Yuan, sp. nov. Figures 2, 3.

MycoBank number: MB 845327.

Diagnosis: *Phaeolus asiae-orientalis* is characterized by pileate to laterally or centrally stipitate basidiocarps, irregular pores 1–2 per mm, abundant vascular elements, mango-shaped basidiospores 6–7 × 3.5–4.2 μm, *L* = 6.34 μm, *W* = 3.82 μm, *Q* = 1.48–1.63 and distribution in Northeast China.

Type: China, Jilin Province, Antu County, the Changbaishan Nature Reserve, dead tree of *Picea*, 21 Sep. 2019, *Y.C. Dai, Dai 20867* (holotype, BJFC032536!).

Etymology: *Asiae-orientalis* (Lat.): referring to East Asia, where the species was found.

Basidiocarps: Basidiocarps annual, pileate to laterally or centrally stipitate on substrate or ground from roots, soft and watery when fresh, and fragile and light in weight when dry. Pilei imbricate, sometimes developing a large number of imbricate, petaloid, or flabelliform and often confluent pilei, circular or semicircular to fan-shaped with irregularly lobed margin, projecting up to 15, 26 cm wide, up to 2 cm thick at the base or center; pileal surface cream, buff, orange when juvenile, pinkish buff, reddish brown to fuscous with age, concentrically zonate with various shades of cinnamon, brown, reddish brown to fuscous colors when fresh, become tomentose to hirsute, umber, rusty tawny to dark brown, indistinctly concentrically zonate when dry; margin acute, incurved when dry. Pore surface greenish yellow, citrine to sulfur yellow, become dark brown when bruised, rusty brown, bay, date brown to purplish chestnut when dry, sterile margin distinct, up to 3 mm wide; pores irregular and labyrinthine, 1–2 per mm; dissepiments thick, entire to lacerate. Context concolorous with a pileal surface, soft corky to fibrous, azonate, up to 10 mm thick. Tubes are pinkish buff to curry yellow, paler than the context, fragile when dry, decurrent, and up to 10 mm long. Stipe central or lateral, sometimes branched, concolorous with a pileal surface, and up to 2 cm long and 1 cm in diameter.

Hyphal structure: Hyphal system monomitic; generative hyphae simple septate, thin- to slightly thick-walled, IKI–, CB–; tissue darkening otherwise unchanged in KOH.

Context: Generative hyphae are hyaline to yellowish, thin- to slightly thick-walled with a wide lumen, occasionally branched, loosely interwoven, encrusted by crystals, and 4.2–15 μm in diameter; gloeoplerous hyphae are present, thin-walled, dark blue in CB.

Tubes: Generative hyphae are hyaline to yellowish, thin- to thick-walled, occasionally branched, subparallel among the tubes, some encrusted by crystals, 3–4 μm in diameter, gloeoplerous hyphae present, thin-walled, and dark blue in CB. Cystidia present, subulate and ventricose or clavate, hyaline, thin-walled, smooth, 65–80 × 10–14 μm, gloeocystidia present, long fusoid, hyaline, thin-walled, dark blue in CB, and 22.5–32.5 × 6.5–8.2 μm. Basidia clavate, bearing four sterigmata and a simple basal septum, and 24–30 × 7.5–9 μm; basidioles dominant, similar to basidia in shape, but smaller. Vascular elements are frequently present in the hymenium.

Spores: Basidiospores are mostly mango-shaped, some ellipsoid, hyaline, thin-walled, smooth, mostly mono-guttulate, IKI–, CB–, (5.5–)6–7(−7.1) × (3.2–)3.5–4.2(−4.5) μm, *L* = 6.34 μm, *W* = 3.82 μm, *Q* = 1.48–1.63 (*n* = 90/3).

Additional materials (paratypes) examined: China, Heilongjiang Province, Tangyuan County, The Daliangzihe National Park, on *Pinus*, August 25, 2014, *B.K. Cui*, and *Cui 11481* (BJFC016723); Wuma County, The Nanwenghe Nature Reserve, on root of *Larix*, August 27, 2014, *Y.C. Dai*, and *Dai 14566* (BJFC017811); Yichun, The Fenglin Nature Reserve, on fallen gymnosperm trunk, 2.VIII.2011, *Cui 9894* (BJFC010787). Inner Mongolia, Arxan, Arxan Forest Park, on the living tree of *Larix gmelinii*, August 24, 2020, *Y.C. Dai, Dai 21647* (BJFC035548). Jilin Prov., Antu County, The Changbaishan Nature Reserve, on the fallen trunk of *Picea*, August 28, 2005, *Y.C. Dai*, and *Dai 7089* (BJFC001617); on rotten *Pinus*, July 28, 1993, *Y.C. Dai, Dai 789* (IFP004023), August 18, 2020, *Y.C. Dai, Dai 21783* (BJFC035684), *Dai 21784* (BJFC035685), and *Dai 21785* (BJFC035686).

**Figure 4:**
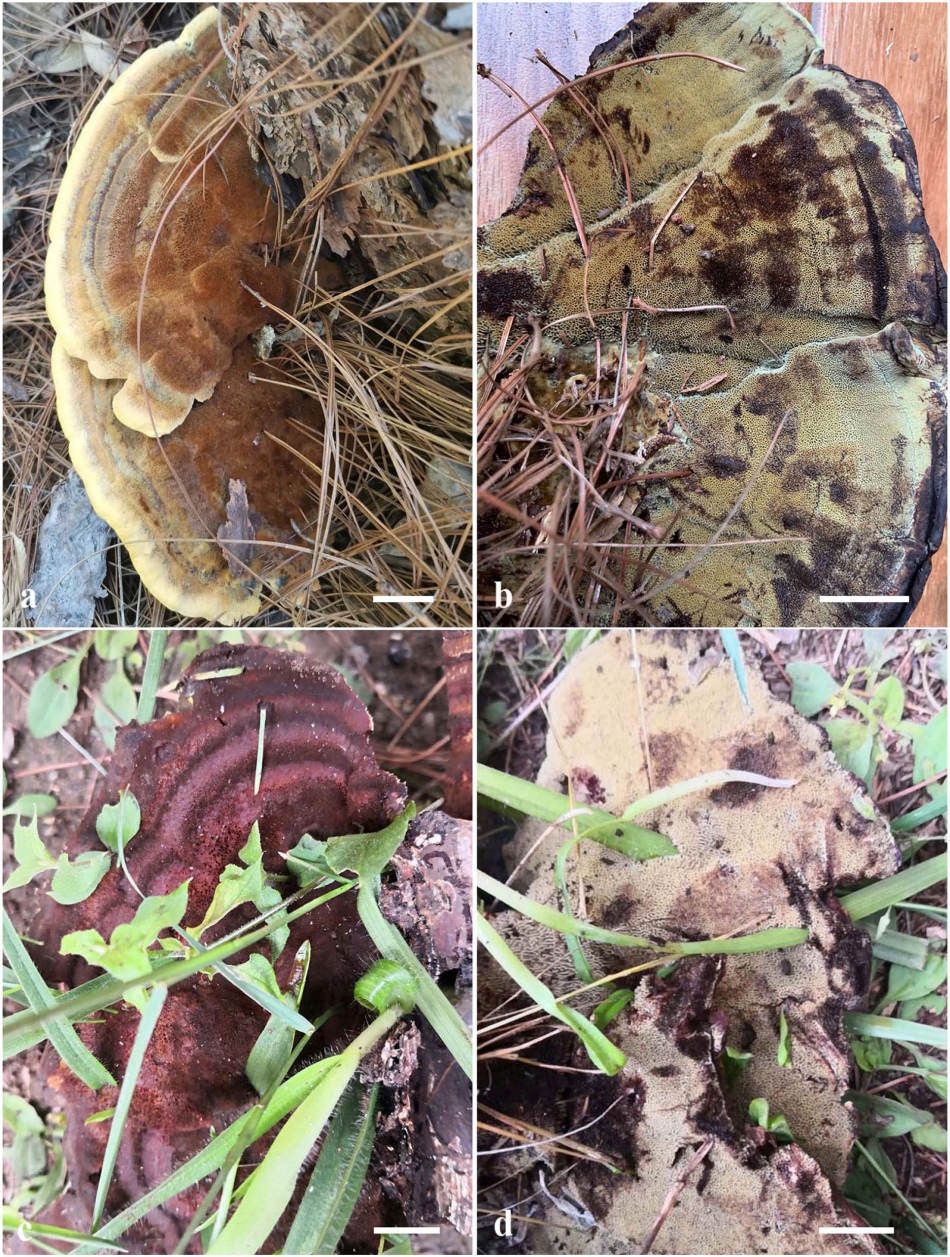
Basidiocarps of *Phaeolus yunnanensis*. **(a,b)** Dai 20426 (holotype); **(c,d)** Dai 22528. Scale bars = 1 cm.

**Figure 5:**
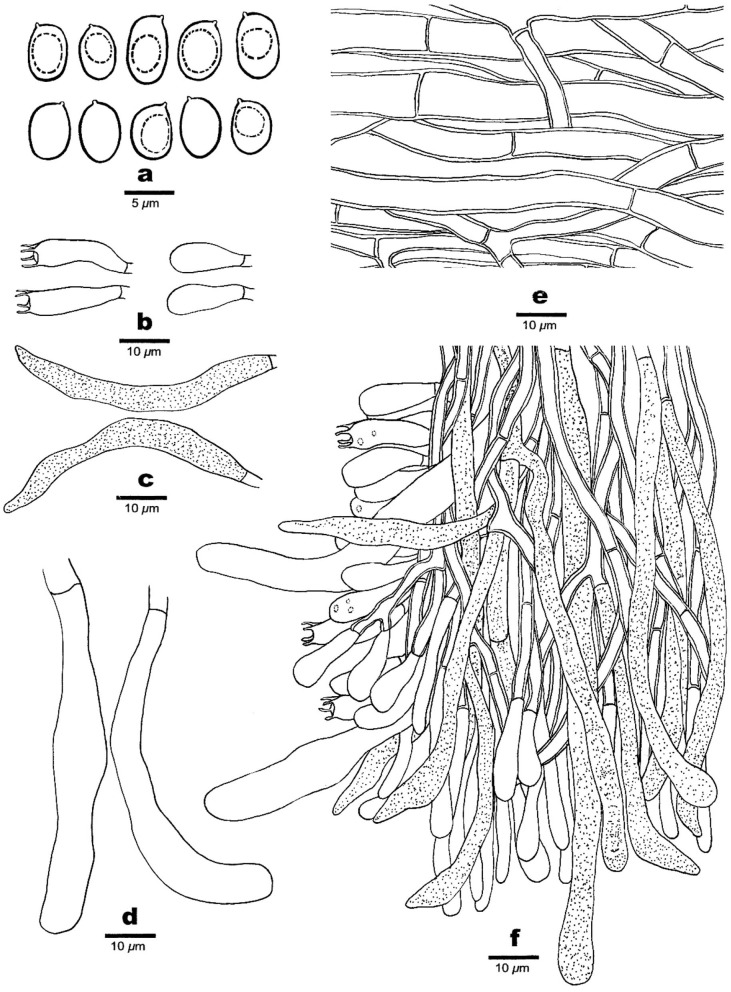
Microscopic structures of *Phaeolus yunnanensis* (drawn from the holotype, Dai 20426). **(a)** Basidiospores. **(b)** Basidia and basidioles. **(c)** Gloeocystidia. **(d)** Cystidia. **(e)** Hyphae from context. **(f)** A section from tube trama.

***Phaeolus yunnanensis*** Y.C. Dai & Yuan Yuan, sp. nov. Figures 4, 5.

MycoBank number: MB 845328.

Diagnosis: *Phaeolus yunnanensis* is characterized by pileate to laterally stipitate basidiocarps, irregular pores of 0.5–1 per mm, absence of vascular elements, ellipsoid to oblong ellipsoid basidiospores 5.5–6.2 × 3.6–4 μm, *L* = 5.85 μm, *W* = 3.83 μm, *Q* = 1.45–1.65 and distribution in Southwest China.

Type: China, Yunnan Province, Yuxi, Xinping County, Longquan Park, the root of *Pinus yunnanensis*, 16 Aug. 2019, *Y.C. Dai, Dai 20426* (holotype, BJFC032094!).

Etymology: *Yunnanensis* (Lat.) refers to the species found on *Pinus yunnanensis* in Yunnan province.

Basidiocarps: Basidiocarps annual, pileate to laterally stipitate, always on wood, soft when fresh, corky to fragile, and light in weight when dry. Pilei applanate, semicircular to fan-shaped with lobed margin, projecting up to 9, 14 cm wide, and 13 mm thick at the base or center. Upper surface buff, fawn to vinaceous brown, concentrically zonate with various shades of buff, cinnamon, vinaceous brown to bay colors when fresh, tomentose to hirsute, indistinctly concentrically zonate when dry; margin acute or blunt, curved inwards when dry. Pore surface cream, greenish yellow to sulfur yellow, slightly glistening, become dark brown when bruised, cinnamon buff, cinnamon, rusty brown, bay to purplish chestnut when dry; sterile margin indistinct to almost lacking; pores irregular and labyrinthine, 0.5–1 per mm; dissepiments thin, entire in juvenile to lacerate to dentate with age. Context concolorous with a pileal surface, azonate, and corky when dry, up to 10 mm thick. Tubes are buff to orange-yellow, paler than the context, fragile when dry, decurrent, and up to 3 mm long. Stipe central or lateral, unbranched, concolorous with a pileal surface, up to 1 cm long and 1 cm in diameter.

Hyphal structure: Hyphal system monomitic; generative hyphae simple septate, thin- to slightly thick-walled, IKI–, CB–; tissue darkening otherwise unchanged in KOH.

Context: Generative hyphae are hyaline to yellowish, thin- to slightly thick-walled with a wide lumen, occasionally branched, loosely interwoven, and 4.5–12 μm in diameter; gloeoplerous hyphae present, thin-walled, and dark blue in CB.

Tubes: Generative hyphae are hyaline to yellowish, thin- to slightly thick-walled, occasionally branched, subparallel among the tubes, 3–4.2 μm in diameter; gloeoplerous hyphae are present, thin-walled, and dark blue in CB. Cystidia present, subulate and ventricose or clavate, hyaline, thin-walled, smooth, and 76–90 × 10–20 μm; gloeocystidia occasionally present, fusoid, thin-walled, dark blue in CB, and 24–31 × 6–7 μm. Basidia clavate, bearing four sterigmata and a simple basal septum, and 25–30 × 6.5–7.5 μm; basidioles dominant, similar to basidia in shape, but smaller. Vascular elements are absent.

Spores: Basidiospores ellipsoid to oblong ellipsoid, hyaline, thin-walled, smooth, mostly mono-guttulate, IKI–, CB–, (5.2–) 5.5–6.2 (−6.5) × (3.3–) 3.6–4 (−4.2) μm, *L* = 5.85 μm, *W* = 3.83 μm, *Q* = 1.45–1.65 (*n* = 90/3).

Additional materials (paratypes) examined: China, Yunnan Province, Chuxiong, The Zixishan National Forest Park, on the rotten root of *Pinus yunnanensis*, 02 July 2021; *Y.C. Dai, Dai 22527* (BJFC037106), *Dai 22528* (BJFC037107). Nanhua County, The Dazhongshan Nature Reserve, on the root of *Pinus yunnanensis*, 15 July 2013, *Y.C. Dai, Dai 13279* (BJFC014768). Yuxi, Xinping County, Longquan Park, on the stump of *Pinus yunnanensis*, July 5, 2021; *Y.C. Dai, Dai 22529* (BJFC037108).

*Other materials studied*.—***P. schweinitzii***. **China**. Xinjiang Autonomous Region, Altay, Burqin County, on the stump of *Larix*, August 19, 2019, *J.Z. Qiu, M542* (BJFC038545). **Belarus**. Brestskaya Voblasts, Belavezhskaya Pushcha National Park, on *Picea*, 19 Oct. 2019, *Y.C. Dai, Dai 21061* (BJFC032720). The **Czech Republic**. Ceské Budějovice, Hluboka, on *Pinus*, Jul. 2004, *J. Vlasák, JV0407/30-H* (dupl. in BJFC038546). **Finland**. Helsinki Botanical Garden, on the root of *Larix*, July 5. 1997, *Y.C. Dai, Dai 2267* (IFP004025); Etela-Hame, Heinola, Kirkonkyla, Papilla, on *Larix*, 11 Apr. 2013, *Haikonen 29485* (H); Tammmela, Mustiala, Maatalousoppilaitos, on *Larix*, April 17, 2016, *Haikonen 30632* (H). **Portugal**. Algarve, Vila do Bispo, Budens, on *Pinus pinea*, 26 Jul. 2018, *Miettinen* (dupl. in BJFC033041).—**“*P. schweinitzii-*1.”** The **USA**. Connecticut, Griswold, Hopeville Pond State Park, on *Pinus strobu*s, 07 Jun. 2021, *D.W. Li*, dupl. *Y.C. Dai, Dai 23689* (BJFC038260), *D.W. Li*, dupli. *Y.C. Dai, Dai 23690* (BJFC038262), *D.W. Li*, dupli. *Dai 23691* (BJFC038263); New Haven, on the ground, October 29, 2021, *D.W. Li*, dupl. *Dai 23688* (BJFC038260). Massachusetts, Boston, Forestry Hill, on *Tsuga*, July 27, 2015, *Y.C. Dai*, Dai 16036 (BJFC020137). Pennsylvania, Wilkes-Barre, Rickettes Glen St. Park, on *Tsuga* roots, July 2003, *J. Vlasák Jr*., JV0307/5-J (dupl. BJFC033026).—***Phaeolus tabulaeformis***. The **USA**. Florida, Sarasota, Water Tower Park, Frisbee golf course, August 13, 2016, Dollinger 873 (dupli. JV and PRM).

## Discussion

Six taxa are detected in the *P. schweinitzii* complex: *P. schweinitzii* sensu stricto seems to be distributed in Eurasia; *P. asiae-orientalis* and *P. yunnanensis* are so far known in Northeast and Southwest China; *P. tabulaeformis* (Berk.) Pat., “*P. schweinitzii-*1” and “*P. schweinitzii-*2” have distributions in North America. *P. schweinitzii* sensu stricto is most probably not distributed in North America, and its previous records in North America (Gilbertson and Ryvarden, 1987) should be analyzed by molecular data.

Twelve names were listed as synonyms of *P. schweinitzii* (Donk, 1974; Index Fungorum Database 2021, http://www.speciesfungorum.org/GSD/GSDspecies.asp?RecordID=121352; MycoBank Database 2021, https://www.mycobank.org/page/Simple%20names%20search); most of these taxa were described from Europe (Donk, 1974), and four were outside of Europe, viz. *Phaeolus amazonicus, P. subbulbipes, P. manihotis*, and *P. tabulaeformis*.

*Inonotus sulphureopulverulentus* P. Karst. was described from Baikal, Russia (Karsten, 1904). Pilát (1936–1942) considered it a species of uncertain identity; Lowe (1956) treated it as a synonym of *P. schweinitzii*. The senior author studied the type (in H), and it is a sterile fragment of basidiocarp. To confirm its identity, new samples from the type locality are needed. However, one sample, SFC2017081018 from Siberia, is *P. schweinitzii*, and our sample BJFC 038545 from the Altay area is also *P. schweinitzii* (Figure 1); the Russian Baikal is closer to Siberia and Altay, and the type of *Inonotus sulphureopulverulentus* is most probably a representation of *P. schweinitzii*.

*Polyporus tabulaeformis* Berk. (1845, type from Augusta, Georgia), *Polyporus spectabilis* Fr. (1851, type from South Carolina, illegitimate) and *Polyporus hispidoides* Peck (1880, type from New York) were described from North America; Overholts (1953) and Donk (1974) treated them as synonyms of *P. schweinitzii*. The former taxon was combined as *Phaeolus tabulaeformis* (Berk.) Pat. by Patouillard (1900). Ryvarden (1977) considered *P. tabulaeformis* to be a synonym of *P. schweinitzii*. However, our phylogenetic analyses show that three taxa of *Phaeolus* exist in North America (Figure 1). Samples from Florida (Dollinger 873 and PhSch) fit the description of *Polyporus tabulaeformis* well (Berkeley, 1845), and they are treated as *Phaeolus tabulaeformis*. Samples from Northeast North America (JV0307/5-J, Dai 16036, Dai 23688, Dai 23689, Dai 23691, DA-38, and FP-102447) most probably represent *Polyporus hispidoides* (type from New York). We treated them as “*P. schweinitzii-*1” because we did not study the type of *P. hispidoides*. Samples from western North America (JLF 5317, JLF 5377, and Mushroom Observer 426394) are treated as “*P. schweinitzii-*2.” We studied two collections of JV0108/104 (from California) and JV0308/64 (from Washington) but failed to extract their DNA, and they were not analyzed in our phylogeny. Samples of JLF 5317, JLF 5377, and Mushroom Observer 426394 from Arizona formed an independent lineage in our phylogeny (Figure 1); they may represent an undescribed taxon. Because our present paper focuses on the Chinese taxa of *Phaeolus*, we are not going to comment much on American taxa.

*Phaeolus schweinitzii* sensu stricto is easily distinguished from the two new species by its bigger basidiospores [6–9 × 4.5–5 μm in Ryvarden and Melo (2017) vs. 6–7 × 3.5–4.2 μm in *P. asiae-orientalis* and 5.5–6.2 × 3.6–4 μm in *P. yunnanensis*].

*Phaeolus tabulaeformis* has pores of 2–3 per mm and basidiospores of 6–7 × 4–5 μm (from Dollinger 873), so its pores are smaller than those in *P. asiae-orientalis* and *P. yunnanensis*, and its basidiospores are wider than those in *P. asiae-orientalis* and *P. yunnanensis*.

“*Phaeolus schweinitzii-*1” resembles *P. asiae-orientalis* and *P. yunnanensis*, but it has mostly oblong ellipsoid basidiospores and scanty vascular elements, while *P. asiae-orientalis* has mostly mango-shaped basidiospores and abundant vascular elements. “*P. schweinitzii-*1” differs from *P. yunnanensis* by longer basidiospores [(6.5–) 6.6–7.5 (−7.7) × (3.5–) 3.7–4.1 (−4.3) μm, *L* = 6.98 μm, *W* = 3.90 μm, *Q* = 1.79 (*n* = 90/3) vs. (5.2–) 5.5–6.2 (−6.5) × (3.3–) 3.6–4 (−4.2) μm, *L* = 5.85 μm, *W* = 3.83 μm, *Q* = 1.45–1.65 (*n* = 90/3)].

*Phaeolus asiae-orientalis* is different from *P. yunnanensis* by longer basidiospores (6–7 × 3.5–4.2 μm vs. 5.5–6.2 × 3.6–4 μm). In addition, vascular elements are abundant in *P. asiae-orientalis*, while they are absent in *P. yunnanensis*.

*Phaeolus asiae-orientalis* and *P. yunnanensis* are common species in both natural and planted coniferous forests in China; they were previously considered *P. schweinitzii*, a forest pathogen in the Chinese forests (Dai et al., 2007; Dai, 2012). The present results demonstrate that the three pathogenetic species of *Phaeolus* exist in China: viz. *P. asiae-orientalis* mostly on *Larix gmelinii, L. olgensis, Picea jezoensis*, and *Pinus koraiensis* in Northeast China, *Phaeolus yunnanensis* usually on *Pinus yunnanensis* in Southwest China, and *P. schweinitzii* on *Larix* in Northwest China. *P. asiae-orientalis* is the cause of butt rot in natural coniferous forests in Northeast China, while *P. yunnanensis* causes butt rot in planted coniferous forests in Southwest China.

*Phaeolus schweinitzii* was also reported in New Zealand and Australia on species of Araucariaceae and Myrtaceae (Cunningham, 1965; Buchanan and Ryvarden, 2000) and in the tropical pacific areas of Hawaii Island on *Acacia* (the Bega, 1979). The taxon differs from *P. schweinitzii* sensu stricto and will be published by another team (personal communication).

*Phaeolus amazonicus* M.A. De Jesus & Ryvarden was described from Brazil, and it differs from the members of the genus in the Northern Hemisphere by its dimitic hyphal structure (Jesus and Ryvarden, 2010). *Phaeolus manihotis* (Heim, 1931) was described from Madagascar. It is different from our two new species by the presence of cuticle or crust at the upper surface and the absence of cystidia (Heim, 1931). *Phaeolus subbulbipes* (Henn.) O. Fidalgo & M. Fidalgo was also originally described from Brazil, and it differs from our new species by its smaller and globose basidiospores measuring 3.5–4.1 μm (Fidalgo and Fidalgo, 1957).

*Phaeolus rigidus* (Lév.) Pat. was described in Java, Indonesia, but according to Ryvarden (1981), it is a synonym of *Trichaptum durum* (Jungh.) Corner.

## Data availability statement

The datasets presented in this study can be found in online repositories. The names of the repository/repositories and accession number(s) can be found in the article/supplementary material.

## Author contributions

YY and Y-CD: design of the research. YY and Y-DW: performance of the research. YY, Y-DW, Y-RW, and MZ: data analysis and interpretation. Y-CD, JV, J-ZQ, D-WL, and H-GL: collection of the materials. YY, Y-DW, Y-CD, JV, and D-WL: writing and revising the manuscript. All authors contributed to the article and approved the submitted version.

## Funding

The research was supported by the National Natural Science Foundation of China (Project Nos. 32161143013, U1802231) and the Second Tibetan Plateau Scientific Expedition and Research Program (STEP, Grant No. 2019QZKK0503).

## Conflict of interest

The authors declare that the research was conducted in the absence of any commercial or financial relationships that could be construed as a potential conflict of interest.

## Publisher's note

All claims expressed in this article are solely those of the authors and do not necessarily represent those of their affiliated organizations, or those of the publisher, the editors and the reviewers. Any product that may be evaluated in this article, or claim that may be made by its manufacturer, is not guaranteed or endorsed by the publisher.
